# Exploring a Road Map to Achieving Tobacco Endgame in sub-Saharan Africa: A Qualitative Study Among Stakeholders From 12 Countries

**DOI:** 10.9745/GHSP-D-24-00351

**Published:** 2026-08-10

**Authors:** Catherine O. Egbe, Senamile P. Ngobese, Arshima Khan, Siphesihle Gwambe, Zinhle P. Ngcobo, Stella A. Bialous

**Affiliations:** a Mental Health, Alcohol, Substance Use and Tobacco Research Unit, South African Medical Research Council, Pretoria, South Africa.; b Department of Public Health, Faculty of Health Science, Sefako Makgatho Health Sciences University, Pretoria, South Africa.; c Department of Psychology, University of Witwatersrand, Johannesburg, South Africa.; d School of Nursing, University of California, San Francisco, CA, USA.; e Center for Tobacco Control Research and Education, University of California, San Francisco, CA, USA.

## Abstract

**Introduction:**

Tobacco endgame seeks to bring an end to tobacco use or drastically reduce prevalence to less than 5%. Discussions about tobacco endgame and the possible strategies to achieve this goal in sub-Saharan African are sparse. This study aimed to explore sub-Saharan African tobacco control stakeholders’ perspectives about tobacco endgame and ascertain what strategies they perceive to be suitable for the region to achieve this goal.

**Methods:**

This qualitative study involved a purposive sample of 29 stakeholders interviewed via online platforms guided by a semi-structured interview schedule. Stakeholders were from academia, civil society, and government departments in 12 sub-Saharan African countries. Interviews were conducted in English or French, transcribed verbatim (those in French were then translated to English), and thematically analyzed with the aid of NVivo version 12 software.

**Results:**

There is support for the adoption of tobacco endgame in sub-Saharan Africa by tobacco control stakeholders in the region due to the negative impact of tobacco on health, the environment, and economy. Proposed endgame approaches for the region were recommended to be Afrocentric and sensitive to cultural and regional dynamics. Stakeholders believed that the success of endgame strategies depends on political will, multisectoral collaboration, availability of resources, buy-in from the public, and monitoring of the tobacco industry. Suggested endgame strategies were categorized into 5 themes: (1) product-focused (less addictive tobacco and regulation of novel products); (2) user-focused (smoke-free generation, cessation support, and promotion of sports); (3) market/supply-focused (licensing of sellers, increase in taxes, control of illicit trade, and alternative income for farmers); (4) institutional structure-focused (tobacco industry monitoring and regulation); and (5) legislation implementation-focused (effective implementation of international treaties aimed at lowering tobacco use prevalence).

**Conclusion:**

There is support for a tobacco endgame in sub-Saharan Africa. Collaboration from various departments/ministries and support from government and the public would be needed to make tobacco endgame a reality in the region.

## Abstract (precis)

Interviews with diverse tobacco control stakeholders from 12 countries in sub-Saharan Africa suggest support for tobacco endgame strategies to end the tobacco epidemic in the region.

## Teaser

**Key Findings:**

- Tobacco control stakeholders in sub-Saharan Africa are supportive of tobacco endgame for the region that moves beyond *controlling* tobacco to *ending* the tobacco epidemic.
- The stakeholders believed that Afrocentric endgame strategies sensitive to culture and countries’ specific contexts are needed.
- Strategies focused on the user, product, supply, institutional structure, and legislation implementation were proposed. Specific examples included finding alternative income for tobacco farmers and cessation support for smokers.
- To ensure successful implementation of tobacco endgame in sub-Saharan Africa, support from governments and the public, along with tobacco industry monitoring, were considered necessary factors.

**Key Implications:**

- Support for tobacco endgame in sub-Saharan Africa exists.
- In fact, stakeholders in the region consider endgame strategies are urgently needed because of the detrimental effects of tobacco use and industry activities targeting young people in the region.

## INTRODUCTION

Tobacco use remains the leading cause of preventable premature death and illnesses globally.^1^ Smokers lose about 10 years in life expectancy,^2^ with an estimated 1 in 2 smokers succumbing to tobacco-related illnesses.^3^ It is estimated that tobacco will claim over 1 billion lives in the 21st century.^4^

In 2019 the prevalence of tobacco smoking in sub-Saharan Africa was 2.9% among women and 17.5% among men, with notable differences by subregion: Central Africa, 1.7% among women and 20.7% among men; East Africa, 3.1% among women and 17.6% among men; Southern Africa, 8.7% among women and 34.7% among men; and West Africa, 1.8% among women and 12.7% among men.^5^

In 2008 the World Health Organization (WHO) introduced the MPOWER measures, aimed at effective tobacco control implementation at the country level^6^^,^^7^:

- Monitoring tobacco use and tobacco control measures
- Protecting people from tobacco smoke
- Offering help to quit tobacco use
- Warning people about the dangers of tobacco
- Enforcing bans on tobacco advertising, promotion, and sponsorship
- Raising tobacco taxes

In countries like Kenya and South Africa, favorable political environments and adequate knowledge provide fertile ground for the implementation of the WHO Framework Convention on Tobacco Control (FCTC) and the MPOWER measures, while in countries like Ghana, Malawi, and Nigeria, prioritization, lack of enforcement of existing tobacco control initiatives, and lack of capacity are major obstacles to effective policy implementation.^8^^,^^9^ Many experts in the tobacco control field believe that forward-thinking strategies that go beyond *controlling* tobacco to *ending* the tobacco epidemic should be explored.

Tobacco control aims to reduce tobacco consumption and its associated health risks by implementing measures that limit its availability, appeal, and use while tobacco endgame envisions a future where tobacco use is eradicated or reduced to minimal levels (currently set at <5%) in society.^10^ Thomson et al.^11^ and Myers^12^ have argued that low prevalence and strong political leadership have translated into the effective implementation of comprehensive tobacco control measures and can serve as critical tools in achieving the tobacco endgame. Policies such as high taxation, advertising bans, and smoke-free laws can limit the availability, affordability, and appeal of tobacco products, creating an environment less conducive to initiation and sustained use. Enhanced public education campaigns and access to cessation support further empower individuals to quit smoking.^13^^,^^14^ By building on proven control measures and integrating bold, transformative approaches, countries can pave the way toward a society with negligible tobacco use and significantly reduced tobacco-related harm.^12^

The concept of tobacco endgame involves implementing strategies aimed at moving beyond tobacco control toward a tobacco-free future wherein the use of commercial tobacco products would either be phased out completely or their availability significantly restricted.^11^^,^^15^ Tobacco endgame has also been defined by some experts as initiatives designed to change or permanently eliminate the structural, political, and social dynamics that sustain the tobacco epidemic so as to achieve an endpoint for the epidemic.^16^ Definitions of tobacco endgame suggest that a key strategy in combatting the global mortality and mobility caused by noncommunicable diseases is through the creation of a world essentially free from tobacco, where less than 5% of people use tobacco.^3^^,^^4^^,^^17^ Endgame strategies vary in method and aspirations, but share 2 underlying beliefs: (1) that the status quo is unacceptable and (2) that reducing tobacco use substantially will require something new, bold, and fundamentally different from existing approaches.^1^ Proposed tobacco endgame strategies include regulating tobacco marketing, prices, and profits; regulating the product to remove or reduce nicotine and toxins; prohibiting tobacco purchasing by those born on or after a certain date; instituting a smoker licensing system;^18^ and eliminating the sale of tobacco products.^19^ Some authors have also proposed a complete ban on cigarettes as a tobacco endgame strategy.^20^ Authors such as McDaniel and colleagues (2015) have categorized proposed tobacco endgame strategies or policies to include focusing on the product, user, market/supply, and larger institutional structures.^15^

The targets of tobacco endgame are expected to be specific and have measurable outcomes that would indicate an end to the tobacco epidemic in a defined geographic area (i.e., country).^21^ Studies have shown generally high public support for most tobacco endgame policies, but support was found to be lower among those who smoke.^22^ Countries such as Bangladesh, Canada, France, Mexico, Portugal, Scotland, Sweden, the United Kingdom, and the United States all have plans to raise a smoke-free generation or drastically reduce or end tobacco use through various policy proposals.^21^^,^^23^ However, prior to the emergence of COVID-19, only 1 country, the Kingdom of Bhutan, previously instituted a complete ban on tobacco.^20^ The national tobacco ban, instituted in 2004, specifically prohibited all sale of tobacco products and banned the use of tobacco products in public areas.^24^ During the peak of the COVID-19 pandemic, Bhutan reversed this ban amid concerns for cross-border smuggling and increased transmission of COVID-19. The ban on production and manufacturing of tobacco is still in place as well as a 100% sales tax on tobacco-related products.^25^

As part of measures to reduce exposure to COVID-19 or reduce the severity of COVID-19 on those who contract the disease, a few countries around the world temporarily banned the sale of tobacco products or restricted their use in public places. Notable among these countries were Botswana, India, and South Africa.^26^^,^^27^ While some researchers have attempted to draw lessons from the outcome of this ban in South Africa for endgame planning,^28^ it should be noted that the temporal tobacco sales ban was neither set to achieve endgame goals^26^ nor did it fit well into the definition of an endgame measure due to the following reasons. The measure: (1) was neither meant to be made permanent nor aimed at completely eradicating tobacco use, based on South Africa’s government documents;^29^ (2) was not proposed as a testing ground for a future endgame strategy in South Africa; (3) did not ban the use of tobacco or nicotine products; (4) was not comprehensive to ensure the entry of other sources of tobacco and nicotine products into the country was controlled or blocked; hence, there were reports of illicit trade during this period; (5) was not systematically introduced as would be expected if it were to be permanent; and (6) was a contingency measure to deal with a novel pandemic that the world had little or no knowledge about at the time it occurred.^30^

Despite governments’ reasons for the ban during the COVID-19 pandemic, studies show that the temporary bans on tobacco sales in several countries created a unique environment that prompted smoking cessation.^31^^–^^33^ These restrictions disrupted access to cigarettes and other tobacco products, forcing or motivating many smokers to abstain, either temporarily or permanently, particularly those who were already considering cessation.^31^ Additionally, heightened public awareness of the risks associated with smoking, such as its link to severe COVID-19 outcomes, further encouraged smokers to prioritize their health.^34^ Studies conducted during this period reported an increase in quit attempts, demonstrating the potential impact of restrictive policies on smoking behavior.^35^^,^^36^

According to WHO, more than 80% of the world’s tobacco users live in low-and middle-income countries (LMICs).^3^ The population in sub-Saharan Africa is particularly vulnerable as it is still in the early stages of a tobacco epidemic and has both the youngest and fastest growing population globally.^37^ The tobacco industry has also increased its marketing and production in the region,^38^^,^^39^ targeting its youthful population. As a result, the sub-Saharan African region is projected to experience one of the highest growth rates in tobacco consumption in the world,^39^ with an estimated smoking rate of 37% by 2025 if current trends are not reversed.^40^ This study therefore aimed to explore the perceptions about the tobacco endgame among tobacco control stakeholders in sub-Saharan Africa as well as the road map and strategies they consider to be suitable for the adoption and implementation of tobacco endgame in the region.

## METHODS

### Study Design and Population

This study employed a qualitative research design using key informant interviews with tobacco control stakeholders working and residing in sub-Saharan Africa, a region that comprises 46 countries with 44 having signed the WHO FCTC.

### Sample and Sampling Technique

We used purposive and snowball sampling techniques to recruit participants. The inclusion criteria were adults aged 18 years or older, resident in one of the 46 sub-Saharan African countries, had at least 12 months working experience in the tobacco control field, and were either a tobacco control advocate, tobacco control researcher, or government official working in tobacco control. In total, we contacted 77 participants from 31 countries (Supplement 1) and conducted 34 interviews. We included data from 29 interviews with participants from 12 countries and excluded data from 5 interviews because the participants said they could not speak about regional issues.

### Data Collection

We conducted the interviews between May and July 2021. We first contacted the participants via email, then through telephone and/or WhatsApp, using initial lists of civil society organizations’ contact persons provided to us by the African Tobacco Control Alliance (ATCA) and the Africa Centre for Tobacco Industry Monitoring and Policy Research (ATIM). ATCA is the regional network of civil society organizations and nongovernmental organizations working in the tobacco control field in the WHO African (AFRO) region with members in 39 countries.^41^ ATIM is a WHO FCTC observatory, which conducts industry monitoring and research across the African continent. ATIM also works with tobacco control researchers, advocates, and governments across the continent.^42^

Individual interviews were mostly conducted via the online platform most suitable for the participant, including WhatsApp (n=1), Zoom (n=9), and Microsoft Teams (n=18), with 1 interview conducted in person. Due to often poor network connections, the interviews were audio-based only (i.e., no video was used). Interviews were semi-structured and guided by an interview schedule developed by the researchers and derived from the key research questions of the study (Supplement 2). The interview questions generally sought to ascertain participants’ understanding of the concept of tobacco endgame, how important it is for Africa to have its own endgame strategies and why, and the strategies participants perceived would help to achieve a tobacco-free Africa.

Participants were interviewed in either English or French and all interviews were audio-recorded irrespective of the platform with which the interview was conducted.

### Data Analysis

We transcribed the audio-recorded interviews verbatim and translated the French transcripts to English. Thematic analysis was conducted with the aid of NVivo version 12 software, following Braun and Clarke’s 6-step framework for conducting thematic analysis (familiarizing yourself with the data; generating initial codes; searching for themes; reviewing themes; defining and naming themes; and producing the report).^43^ Suggested endgame strategies were grouped by the categories published by McDaniel et al.^15^ and Puljević and colleagues^21^ (product focused, user focused, market/supply focused, institutional focused). However, we were also open to identifying new endgame strategies differing from known strategies in the field, hence the addition of new subthemes to the predefined categories as well as a new category (legislation implementation-focused) emerging from the data.

COE, AK, ZN, and SG initially coded a sample of transcripts, after which, AK, ZN, and SG completed the analysis. COE and SPN then reviewed the completed analysis. SAA helped with resolving disagreements with interpretation and categorization of themes during this process.

### Reflexivity

As tobacco control researchers, it is important to acknowledge that our own professional background within tobacco control may have shaped our interpretation of the data. To mitigate this, we regularly held feedback meetings to seek alternative and consensus perspectives on the data. All authors accepted the final categorization of the themes.

### Ethical Considerations

Ethical clearance to conduct this study was obtained in April 2021 from the South African Medical Research Council’s Human Research Ethics Committee (Protocol ID: EC014-4/2021). Information sheet/consent forms were used to communicate details about the study in English and French. Each participant was requested to read through the information sheet/consent form, sign, and return the signed form to the project team as confirmation of their intention to voluntarily participate in the study. Participants were informed about their right to discontinue or withdraw from the study at any time and were assured of anonymity and confidentiality throughout the study. Participants were also informed that all information shared or published would be de-identified except for country names. Code names were used to ensure anonymity. Audio recordings and transcribed data have been stored in password-protected laptops that are only accessible to the principal investigator and coauthors.

## RESULTS

The distribution of the participants by country, sex, and occupation is presented in Table 1. Most of the participants were men (about 76%) and worked for nongovernmental organizations (72%).

**TABLE 1.** Sociodemographic Characteristics of Participants (N=29)

**Characteristic**	**No. (%)**
**Country**	
South Africa	5 (17.2)
Nigeria	5 (17.2)
Kenya	4 (13.8)
Ghana	3 (10.3)
Benin	2 (6.9)
Senegal	2 (6.9)
Burkina Faso	2 (6.9)
Ethiopia	2 (6.9)
Togo	1 (3.4)
Zimbabwe	1 (3.4)
Uganda	1 (3.4)
Côte d’Ivoire	1 (3.4)
**Sex**	
Male	22 (75.9)
Female	7 (24.1)
**Occupation**	
Nongovernmental organization	21 (72.4)
Governmental department	4 (13.8)
Research/academia	4 (13.8)

We grouped the findings into 5 categories with their associated themes and subthemes (Table 2):

**TABLE 2.** Themes and Subthemes From Participant Interviews Related to Tobacco Endgame in sub-Saharan Africa, by Main Category

**Categories**	**Themes**	**Subthemes**
Perception of the endgame concept and its importance for sub-Saharan Africa	Defining the endgame concept	Tobacco-free Africa End of tobacco use (tobacco use/products being nonexistent/zero-smoked cigarettes) Reduced tobacco farming Endgoal of tobacco control
		Reining in the tobacco industry End of the manufacturing and sale of tobacco in sub-Saharan Africa
	Importance of endgame for the region	Negative impacts of tobacco Economic impact Health impact Environmental impact
		Tobacco industry targeting Africa
Perspective on sub-Saharan Africa’s approach to tobacco endgame	Afrocentric strategies sensitive to culture and regional dynamics African Charter on Tobacco Endgame^a^ Country-specific endgame strategies (national strategies culled from the regional strategies) Clearly defined and systematically organized endgame goals	
How to implement tobacco endgame in sub-Saharan Africa	Education and awareness campaigns Use of new media and the entertainment industry for raising awareness Including traditional and religious leaders in advocacy for endgame Conducting reliable and robust local research	
Facilitators of the adoption and implementation of tobacco endgame in sub-Saharan Africa	Effective education and awareness campaigns Political will Multisectoral collaboration Availability of funds and other resources Buy-in from the public Tobacco industry monitoring	

a A regional position or white paper focused on how to end commercial tobacco use in Africa.

- Perception about the concept of tobacco endgame and its importance for sub-Saharan Africa
- Perspectives on how the region should approach tobacco endgame
- Road map to the implementation of tobacco endgame in the region
- Facilitators to the adoption and implementation of tobacco endgame in the region
- Suggested tobacco endgame strategies for the region

There was a general perception that effective implementation of the WHO FCTC in controlling tobacco is an essential road map toward tobacco endgame in sub-Saharan Africa because the strengthening of tobacco control measures would adequately prepare countries for the adoption of endgame strategies.

### Perceptions About the Tobacco Endgame Concept

#### Definition of the Tobacco Endgame Concept

Participants’ definitions of the tobacco endgame were grouped into 2 main themes: tobacco-free Africa (end of tobacco use) and ending the tobacco business. Findings show that some participants already envisioned a tobacco-free future for the African region and their country. Slogans mentioned by a participant to express this vision included “tobacco-free Africa” and “tobacco-free Kenya.”

*We talk about the tobacco-free Africa. In Kenya, we say tobacco-free Kenya, meaning that we are looking forward to a situation whereby we will not be having tobacco at all. And that is something … the vision is very valid*.

For other participants, tobacco endgame meant the end of tobacco use in all its forms and the end goal of tobacco control. As one participant from Côte d’Ivoire explained:

*Tobacco endgame would assume that we will arrive at a point whereby the consumption of tobacco in its different forms that we know will no longer exist, and that more and more the population, especially young people, will turn away from tobacco in all its forms. When I talk about all its forms, as for example nowadays, in terms of cigars, cigarettes, cigarillos, tobacco in the forms of new products such as shisha and others, in all its forms it will end.*

However, other participants understood the concept to mean a way to rein in or end the tobacco industry, which was seen as a business that propagates the tobacco problem. This implies an end to the manufacturing, sale, use, and farming of tobacco. One participant from Ethiopia described:

*Tobacco endgame, actually, you know I have one slogan … when I am talking about the industry, this is the industry that should die. Tobacco endgame is to me to finish the tobacco business and to make the whole globe tobacco free, so that means there isn’t any tobacco issue or product in the whole earth.*

#### Importance of Tobacco Endgame

Participants mentioned that tobacco is negatively impacting the sub-Saharan African region. Three subthemes emerged as to why the complete eradication of tobacco is important for the region: the tobacco industry’s targeting of Africa, the negative impact of tobacco, and the region’s fragile health care system.

Participants thought the tobacco industry was especially targeting young people in the region and that ending tobacco use would protect the region’s youth. As one participant from Nigeria explained:

*[We need to end tobacco use]* … *because the tobacco industry is targeting the youth and they want to make Africa their major market.*

The negative impacts of tobacco were grouped into the economic, health, and environmental impact of tobacco. The **economic impact of tobacco use** on health and health systems in sub-Saharan Africa was of concern to participants, including the financial cost of treating tobacco-related illnesses, which burdens the already weakened economy, as well as the strain on the fragile health system. A participant from Ghana stated:

*… it has become so imperative because we are a struggling and a developing country and, therefore, we do not have the luxury of better health care, we do not have the luxury of a better condition to manage crises as compared to the Western world and that is why it is very important. It is a matter of urgency that we step up the campaign and this endgame campaign, in order to prevent the continent from getting into a situation of dire health and economic situation.*

A participant from Kenya described the net financial loss that governments suffer as a result of tobacco when compared with the revenue generated from the industry:

*The tobacco industry will talk about taxes that they bring to a government, but when you work it out, the government spends more in treating tobacco-related diseases, and also the vulnerability that is brought as a result of tobacco use.*

Participants also mentioned that tobacco leads to serious **health implications** that are preventable, which then puts additional burden on the already burdened health system, as seen especially during the peak of COVID-19. They noted how the region is facing multiple challenges with ever-increasing communicable and noncommunicable diseases.

*Looking at the health care system, the health infrastructure, you realize that in Africa, we are still battling with TB and HIV/AIDS and malaria, we are still battling with them. COVID has exposed us even more, we don’t have the health facilities. It’s not easy to admit as citizens, we don’t have it.* (Participant from Ghana)

*We are concerned about this [tobacco] because of the impact it has on health, and on the health systems. Mind you, in Africa, most countries, their health systems leave much to be desired. You know, they are not well resourced in terms of person power, they are not effective and efficient, they don’t have the systems, they don’t have the tools to enable them to run health systems in an effective manner. And so, we cannot afford unnecessary diseases affecting the people who are really sick, with the tobacco-related diseases affecting the systems, health systems unnecessarily.* (Participant from South Africa)

The **environmental impact of tobacco use,** which includes pollution of the environment, was also mentioned as a reason why endgame strategies are important to implement in sub-Saharan Africa.

*Tobacco pollutes the environment, especially in agriculture. They use several chemicals like pesticides, and they will have an impact in the future. They are cancerous, they will cause cancer in the future and such chemicals are transferable in nature. They will transfer from the soil, water, and other vegetables, then they will be eaten by the people, you know, it’s a continuum.* (Participant from Ethiopia)

### Perspectives on the Region’s Approach to Tobacco Endgame

Participants were asked if they believed that unique strategies were needed to end tobacco use in Africa and which strategies were needed. They suggested strategies that include being ‘Afrocentric’ and sensitive to culture and regional dynamics or to be in the form of an Africa charter on tobacco and health. They also suggested strategies should be country specific, that they should have clearly defined goals, and that they be systematically organized.

Participants proposed that endgame strategies for the region must be **Afrocentric**, meaning that they must cater to the African context and be sensitive to African culture and regional dynamics given the diverse nature of the region. A participant from Ghana explained:

*Like I said earlier on, every continent has its dynamics, Africa has its dynamics, and even in the region of Africa, the subregional countries have their own dynamics, southern Africa, eastern Africa, northern Africa, western Africa, central Africa, even though it all belongs to one region, there are dynamics, you get it. That is where strategies are very key and strategies that are to be implemented by the endgame to achieve this where we will end tobacco in Africa, must also be looked at based on the subregional dynamics and that is where strategies can be implemented effectively to achieve the results. That is why I said if Africa is able to have its own strategy it must be even disseminated to subregions, and each nation will be able to have its own strategies out of those ones.*

A participant from Nigeria further proposed an **African charter** for tobacco and health that would encompass a regional position on tobacco:

*To actually have an African position on tobacco and health. We do need to have like an African Charter of Human Rights, for instance, which flowed directly from the UN Charter on Human Rights. Can we begin to work for an issue position resolution on tobacco use on the continent?*

Participants posited that countries within sub-Saharan Africa, although similar, are unique because some countries are dependent on tobacco farming. They mentioned that while a regional framework for tobacco endgame is possible, the same endgame strategies may not be applicable for all countries within the region. Such frameworks should take into consideration whether the country grows, manufactures, or imports tobacco. **Country-specific endgame strategies** were therefore proposed.

*I am not too sure I can use the word unique. However, I would want to use the word; strategies that respond to the needs, maybe that’s where unique comes in—unique to the environments of each country—I can put it that way. Because countries differ politically, countries differ in the way they are structured economically, and the framework perhaps is the same. We can have the same framework, but what could differ is how the strategy is executed, because each strategy has to respond to a particular environment. Of course, there would be similarities, but the strategy is the policy, and if we look at the policy, and you then have your regulations. If you look at regulations, you need to have all those functional elements of policy execution and of course that could differ, and then of course the implementation will differ country by country.* (Participant from South Africa)

*I think it’s going to be country-dependent based on how those countries are, how they prioritize tobacco in the first place, and what they do in the area of tobacco control. Are they grower? Are they manufacturers or are they just importers? It’s a number of things that needs to be considered, not just one blanket solution.* (Participant from Nigeria)

*Ethiopia has a culture that discourages this product, that means it is going to violate the culture of the community if it is expanding in the country … maybe we can use cultural means to implement the endgame.* (Participant from Ethiopia)

Participants also suggested that strategies must have **clearly defined goals and be systematically organized** to ensure the effectiveness of such measures.

*To reduce tobacco use to the barest minimum or even stop it, it has to have a coordinated approach. It has to be a systematically organized strategy. It’s not just to try this today. So you need a coordinated endgame strategy to be able to achieve the goal that you need. If not, we’ll just be doing all sorts, and I don’t know whether it will be effective. So, for effectiveness, then we need the endgame strategy.* (Participant from Nigeria)

### Road Map to Tobacco Endgame Implementation

Findings from this study revealed that while embarking on tobacco endgame, some important steps to take to ensure its success include public awareness programs, use of new media and the entertainment industry to raise awareness, engagement of traditional and religious leaders in advocacy, and conducting reliable and robust local research to inform policies.

**Public awareness** (through public education and communication) was seen as a type of strategy that sub-Saharan Africa can use to educate people about the dangers of tobacco and the importance of health, and to decrease the consumption of tobacco in the region.

*… there should be massive education on the continent about the dangers of tobacco use.* (Participant from Nigeria)

Participants stated that these campaigns should be targeted at adolescents and women due to the increasing tobacco use among these populations.

*… more and more we really note that there are young girls who are now subscribed to tobacco … if we develop information and education programs and if we involve the grouping of women’s training and youth movements, I think that we can actually succeed in redressing the use of tobacco although it is very difficult to eradicate strictly the use of tobacco in our countries.* (Participant from Senegal)

The **use of new media** and the entertainment industry for awareness-raising, as well as ensuring smoke-free movies, were also mentioned as a strategy to discourage smoking in the population toward ending tobacco use.

*… use the entertainment industry to set a template for, to set a standard, which you get to stop smoking in movies, and for celebrities to now also serve as role models to discourage smoking.* (Participant from Nigeria)

Additionally, participants suggested that there is a need to include **religious and traditional leaders** in advocacy to end tobacco use given the high regard Africans have for such institutions.

*Africans are people who actually uphold their culture in a high esteem, and I believe that if we are able to get our messages across various religious leaders and traditional authorities, where we are able to make them understand and for them to also buy into our campaign, when the messages and the advocacy and education is coming from the religious leaders, is coming from the traditional authorities, where people hold in high esteem. I believe that it is one strategy that can help a lot in order for us to be able to end this tobacco consumption.* (Participant from Ghana)

Participants also emphasized the need for more **reliable and robust local research** within the region to inform endgame policies. According to one participant from Nigeria, there is a need to develop and rely upon data generated from African countries to inform solutions to regional problems:

*Africa, we need our own material … we need to start telling our own stories, not using the stories of beyond borders, to try solving a very unique matter on our own soil, on our land.*

### Facilitators to Tobacco Endgame Adoption and Implementation

Participants proposed that effective education and awareness, political will, multisectoral collaboration, availability of funds and other resources, buy-in from the public, and tobacco industry monitoring could help achieve successful tobacco endgame adoption and implementation in sub-Saharan Africa.

The participants mentioned that **education and awareness** must be hinged on reliable and unbiased data and media campaigns, which are more likely to catch the attention of policymakers.

*So I believe that … carrying out studies and working with the national statistical agency of the scientific and demographic of the country which regularly conducts research … will be possible for us to tell the authorities that govern us, for example, what is needed for each area.* (Participant from Senegal)

**Political will** from regional and subregional bodies and national governments, as well as decision-makers, was also identified by participants as one of the main factors that would fast-track tobacco endgame in the region.

*Something that also came to me is government commitment or political will. I really think that is the most important factor, because once these things are in place, for example, AU [African Union] should pick this up as top priority and cascade it down to other countries so they can also take this as a priority. There will be an endgame in a short while because the commitment will be there.* (Participant from Nigeria)

Another factor mentioned was **multisectoral collaboration**. Participants suggested that a unified approach among all stakeholders and government departments will help end tobacco in sub-Saharan Africa.

*No human work is perfect, but I think that the will counts. If the will is there at the regional and national level, even the involvement of all actors such as civil society and others, if everyone gets on with it, nothing is impossible. We often say that together we go far, so if we get together, it is obvious that we will go far.* (Participant from Côte d’Ivoire)

Some participants emphasized the importance of adequate **human and financial resources and buy-in from the public and the media** to determine the success of tobacco endgame in sub-Saharan Africa.

*The mobilization of resources … I am talking about human resources. It is necessary that there are more actors who are interested in the theme. We also need financial support to always innovate in terms of actions.* (Participant from Côte d’Ivoire)

*… media buy in. If there’s media interest, if the media is ready to talk and write about it, that is a good factor.* (Participant from Ghana)

Lastly, **tobacco industry monitoring** was mentioned as an important factor that will also determine the success of tobacco endgame in the region. According to some participants, most challenges against the endgame will likely stem from tobacco industry interference.

*We need to continuously monitor the tobacco industry … So the tobacco industry is working 24/7, so we also need to be watching them 24/7 and doing something about it and building operations including the media that will continue to embarrass the industry, to put the information out there to contradict the lies that they are putting out there.* (Participant from Uganda)

*And of course, that goes without saying that tobacco interference should be stopped or reduced to the barest minimum. These factors will go a long way in helping tobacco control and finally ensuring that we prevent younger people, non-smokers from taking on tobacco smoking.* (Participant from Nigeria)

### Strategies to Achieve Tobacco Endgame

We used the 4 groups of policies with potential to achieve a tobacco endgame published by McDaniel and colleagues^15^ as well as Puljević and colleagues^21^ to categorize participants’ suggested endgame strategies for sub-Saharan Africa. The 4 groups include policies focused on the product, user, market/supply, and institutional structure. Not all specific strategies identified by McDaniel and Puljević under each of the 4 categories were identified in this study; however, additional strategies suggested by the participants that related to the 4 policy categories were added to those categories. In addition, one new policy category was added focused on legislation implementation (Table 3).

**TABLE 3.** Strategies Proposed by Participants to Achieve Tobacco Endgame in sub-Saharan Africa, by Policy Category

**Policy Category**	**Proposed Strategies**
Product-focused	Making tobacco less addictive Regulating novel products (e-cigarettes and hookah)
User-focused	Instituting smoke-free generation policy Increasing cessation support efforts: cessation services in health care facilities and toll-free national quit line Exploring local remedies for treating nicotine addiction Using sports to promote an end to tobacco use
Market/supply-focused	Licensing tobacco sellers Increasing tobacco taxes Controlling illicit trade Finding alternative sources of income for tobacco farmers
Institutional structure-focused	Monitoring and regulating the tobacco industry
Legislation implementation-focused	Implementing the WHO Framework Convention on Tobacco Control Signing and implementing the Protocol to Eliminate Illicit Trade in Tobacco Products

#### Product-Focused Strategies

Endgame strategies focused on tobacco and related products included a reduction in the addictiveness of tobacco products and the regulation of novel products like e-cigarettes and reemerging tobacco products like hookah. Participants saw these as policies with the potential to end tobacco use. Two participants from South Africa explained:

*So I think for me one strategy that we can use, that I really liked when I heard about, is making tobacco less addictive. Reducing nicotine content and move it to less addictive rates. Because for me stopping it completely will be difficult, so progressively cutting down on the addictiveness of tobacco over time, that is what Africa should be doing.*

*Regulating e-cigarettes and all kinds of novel tobacco products, and that’s going to have a big impact on the e-cigarette market.*

#### User-Focused Strategies

In addition to the regulation of products, participants mentioned the need for a **smoke-free generation** policy, which entails putting in place legislation where children born in, or after, a particular year can be prevented, by law, from buying tobacco products.

*Part of one of the endgame strategies is the so-called ‘smoke free’ generation. I think in the Netherlands, they had the strategy, if that’s still the case, [that] everyone born in the 21st century, not a single one we want to see smoke. So what that means is that in 50, 60, 70 years’ time, the smoking generation would have died out.* (Participant from South Africa)

The importance of having **cessation services** in health care facilities and a national quit line were also noted as a strategy to end tobacco use in sub-Saharan Africa.

*The other strategy could be cessation. Once they have the addiction … they have a right to have this intervention to be free from this addiction. So the service should be integrated within the health services, for instance, in the primary health care system.* (Participant from Ethiopia)

*I think we can do more in the African region as a whole. The cessation and the addiction part is something we need to prioritize over time, but we are already seeing other countries start, and those services will be available.* (Participant from Nigeria)

Another user-focused strategy proposed was exploring **local remedies** for treating nicotine addiction.

*There should be local-based measures, especially, as I have mentioned, using specialized local available material to heal that [nicotine] addiction. So overall implementation of cessation services which are local to the country or continent is very, very important.* (Participant from Ethiopia)

Lastly, participants were of the view that when young people are encouraged to take up **sports** and join **fitness clubs**, this could help to dissuade them from using tobacco**.**

*I believe that sports can be a means for us to fight against tobacco … I believe that if we ensure that at an early age, citizens are introduced to sports and citizens have the opportunity to subscribe to sports, even if there are not enough of them at the moment, and if there are opportunities that are put in place, I believe that this will be important. In Senegal we notice more and more fitness clubs where there are people, especially young people and adults, who now subscribe to sports. Personally, I believe that in this area of the development of sports practice we can also contribute effectively because we remain positive in the fight against tobacco.* (Participant from Senegal)

#### Market/Supply-Focused Strategies

A participant from South Africa mentioned that tobacco products like hookah are available everywhere and there is need to have control over this by licensing sellers:

*Think about hookah or the hubbly bubbly, it’s too freely available. There are no licenses, there is no control over it, and also it is an addiction concern.*

Some participants believed that if **tobacco taxes are increased** significantly, this could help to reduce tobacco use drastically.

*So I believe that if we continue to increase the taxes, I mean who knows, we can reduce tobacco use to decrease to maybe the single digits, below 10%.* (Participant from South Africa)

The **control of illicit trade** was described as an important strategy to reduce the availability of tobacco products. For example, a participant from Kenya explained that the track-and-trace system introduced to fight against illicit trade in Kenya has proven to be effective:

*The Kenya Revenue Authority, fighting illicit trade, introduced a track-and-trace program. That is a win. It is to ensure that every tobacco that is sold in the country is a legal product. It has not been snuck through the borders or it has not been taxed. So it is generally a way or a mechanism that has been created by the Kenya Revenue Authority that ensures that we, that all the tobacco that is sold in the country, has been taxed, is here legally, and that is important.*

Some participants suggested that any endgame strategy planned for sub-Saharan Africa must include as a key component addressing how to help **tobacco farmers transition into farming other crops** to protect their means of livelihood.

*… there won’t be any endgame in Africa without the policy to move tobacco farmers from cultivating tobacco to other wholesome products [to preserve] their livelihoods that are already dependent on tobacco farming in Africa. So that also should be a key component of our endgame strategy.* (Participant from Nigeria)

*We, in order to implement this endgame policy and to bring it about successfully, need to encourage and, in fact, get people to support and sponsor farmers to change from tobacco into more profitable crops. There are many.* (Participant from South Africa)

#### Institutional Structure-Focused Strategies

Institutional structure-focused strategies mentioned by the participants include **monitoring and regulation of the tobacco industry**. Some participants noted that the tobacco industry continues to manipulate laws and exploit the region for profit; hence, it is important to continuously monitor this industry to address tobacco use in the region.

*And so, the industry must be regulated, the industry must be monitored, the industry must be forced to report, even including their revenues. If their revenues keep escalating, it means something is wrong around tobacco control interventions. It means they are still getting more out of a product killing people, you see. So the industry is a major threat and what we need is strong and robust regulation of the industry.* (Participant from South Africa)

*If we can continue to monitor what the industry is doing, we will be able to respond. I think that will make a difference for Africa.* (Participant from Uganda)

#### Legislation Implementation-Focused Strategies

For some participants, endgame strategies were important but not a matter of urgency currently because, according to one participant, there is need to **lower the prevalence of tobacco use** in the region before an endgame strategy is considered. Lowering the prevalence of tobacco use was considered a prerequisite for sub-Saharan African countries to embark on an endgame path. Stakeholders also proposed that reducing tobacco use can be achieved by effective implementation of international treaties like the WHO FCTC and the Protocol to Eliminate Illicit Trade in Tobacco Products,^44^ as well as national tobacco control laws. Some participants also believed that these treaties in themselves, if effectively implemented, could end tobacco use in the region.

*You only start thinking of the endgame strategy if your smoking prevalence has decreased a lot, and is becoming very low, and starting so low that you start thinking about the 5%. If your smoking prevalence is still 15 to 18%, as is in South Africa’s case, I don’t think it’s the right time to be thinking about endgame strategies as yet.* (Participant from South Africa)

*We fully implement all the provisions of the FCTC, the provision of our tobacco control laws. So far this is how I see the end of tobacco, the endgame in Africa.* (Participant from Senegal)

*I think one of the key things to do in South Africa is to try and eliminate illicit trade, but not only just signing it [the protocol] because I think sometimes countries, or people in general, will just get into the habit of just signing things and putting them on paper, but not implementing them. So it is not only the matter of signing the protocol but actually try and implement, so things like the illicit trade and tax, make sure that we implement.* (Participant from South Africa)

## DISCUSSION

To the best of our knowledge, this is the first study conducted to date that explores tobacco control stakeholders’ perceptions about the concept of tobacco endgame in Africa. The stakeholders’ understanding of endgame, though varied, were in line with previous definitions^15^ of moving away from controlling tobacco use toward ending it completely and envisioning a tobacco-free future for their country and the region.

Discussions and policies are already being put in place to move tobacco control policies and interventions toward an endgame plan in some parts of Europe and the United States. For example, in 2021, the European Union announced a tobacco-free generation for Europe as part of Europe’s Beating Cancer Plan.^45^ In November 2024, the United Kingdom announced a bill to create a smoke-free generation and phase out the sale of tobacco products to anyone aged 15 years or younger in 2024.^46^ However, there is no data showing that any country in sub-Saharan Africa has considered a tobacco endgame vision.^21^ This study showed that tobacco control stakeholders in sub-Saharan Africa do believe that tobacco endgame strategies are important because of the negative effects that tobacco has on the environment, people’s health, and the economy. This importance is especially key given that the region was projected to experience one of the highest increases in tobacco use prevalence by 2025 if no action was taken.^40^

Findings from this study show that the interviewed stakeholders from sub-Saharan Africa were particular about the endgame strategies that would be suitable for the region. They believed that Afrocentric approaches should be particularly designed to fit the context, culture, and regional dynamics of Africa as well as include the involvement of religious and traditional leaders who are highly respected public figures in the region. This finding supports the assertion by Malone et al. that for endgame approaches “different solutions will prove possible in different places and may unfold in unique ways.”^16^^(p43)^ Thus, tobacco endgame planning must consider the jurisdiction-specific tobacco control context.^47^

Our findings also reveal that, according to the interviewed stakeholders, success of the tobacco endgame in sub-Saharan Africa depends on various factors, from political will to public support. Political will is instrumental in establishing legislation and successfully implementing tobacco endgame strategies.^11^ In Uruguay, key politicians supported tobacco control, leading to a decrease in prevalence by 7% in 3 years.^11^ Furthermore, in 2018 6 countries, including Canada and Sweden, introduced government-endorsed endgame goals.^48^ Research has shown that the public does support efforts to decrease or end the use of tobacco if properly defined and communicated by the government.^15^

As previously mentioned, McDaniel et al.^15^ and Puljević et al.^21^ described some policies that have the potential to achieve the endgame, grouped in 4 categories: product-focused, user-focused, market/supply-focused, and institutional-focused. In addition to these key policies, this study also recommended a legislation implementation-focused approach. This approach, which should primarily focus on effective and full implementation of the WHO FCTC as well as signing and implementing the Protocol to Eliminate Illicit Trade in Tobacco Products, should focus on strengthening national laws through effective implementation plans aimed at reducing prevalence—seen by some stakeholders as a prerequisite for embarking on an endgame plan. This is particularly important for sub-Saharan African countries with high prevalence of tobacco use like South Africa.^49^

While some stakeholders in this study considered the smoking prevalence of many sub-Saharan African countries too high for the consideration of embarking on an endgame approach, it should be noted that there are many countries in the region that already have a low prevalence of less than 10% and could be ready to take on an endgame approach to end tobacco use. Examples include Ethiopia and Tanzania, with prevalences of 5%^50^ and 8.7%,^51^ respectively. These and many other sub-Saharan African countries with low prevalence could adopt endgame strategies, provided there is wide public understanding and support across various groups, and use of evidence-based strategies.^11^^,^^52^

Important for consideration is the fact that even in countries that have embarked on endgame, tobacco endgame measures are being supported by evidence-based conventional tobacco control measures. This implies that tobacco control and endgame measures are not mutually exclusive and should be implemented hand in hand, as weak tobacco control implementation would be inimical to the effective implementation of tobacco endgame measures.

Our study has shown that despite the different elements needed by different sub-Saharan African countries to possibly embark on an endgame journey, there is support for tobacco endgame among stakeholders of tobacco control in the region. However, further research is needed to explore the general public’s understanding of the endgame concept as well as its purpose to curb the tobacco epidemic in the region. Such insights must include spreading awareness that tobacco control and tobacco endgame strategies are not mutually exclusive and that an effective tobacco endgame policy must begin with strong tobacco control laws that are effectively implemented.

### Limitations

This is a qualitative study that generated data from key informant interviews. While consistent with achieving our aim, it limits our ability to generalize our findings to the entire tobacco control stakeholder community in sub-Saharan Africa. The stakeholders in the field of tobacco control, however, were carefully selected to cut across at least one-third of the countries in sub-Saharan Africa and various sectors including academia, advocacy, and government. The selection criteria also ensured that these stakeholders have worked in the tobacco control field for at least 1 year prior to the study. Further research should include a higher number of stakeholders in a quantitative survey to ascertain if this support is widespread.

## CONCLUSION

This qualitative study reveals that there is support for tobacco endgame among stakeholders in sub-Saharan Africa. Tobacco control stakeholders also believe endgame strategies are urgently needed because of the detrimental effects caused by tobacco use and industry activities targeting young people in the region. Proposed endgame measures that could facilitate the eradication or significant decrease in tobacco use in the region were recommended to be Afrocentric, sensitive to the cultural and regional dynamics, systematic, and with clearly defined goals. For endgame strategies to succeed in sub-Saharan Africa, collaboration among various government departments, stakeholders, and support from the public was recommended. Product, user, market/supply, and institutional structures, as well as legislation implementation-focused policies, were suggested. Findings from this study can be used to continue the conversation and inspire regional and subregional considerations for tobacco endgame in the sub-Saharan Africa region.

## Notes

**Peer Reviewed**

**First Published Online:** March 4, 2026.
